# ADAPTING A CAREGIVER TRAINING PROGRAM TO SUPPORT EQUITABLE IMPLEMENTATION DURING CARE TRANSITIONS

**DOI:** 10.1093/geroni/igae098.1424

**Published:** 2024-12-31

**Authors:** An Nguyen, Pamela Roberts, Leslie Davidson

**Affiliations:** Cedars-Sinai Medical Center, Los Angeles, California, United States; Cedars-Sinai Medical Center, Los Angeles, California, United States; The George Washington University School of Medicine and Health Sciences, Washington, District of Columbia, United States

## Abstract

Transitions in care (TOC) interventions can improve transitional care processes and reduce hospital readmissions in the general older adult population. People living with dementia (PLWD) are often excluded from TOC intervention studies due to the potential for cognitive impairments to interfere with the intervention’s theorized mechanisms of change. Given that PLWD experience worse health outcomes following hospitalization and more frequent, costly transitions in care, there is an urgent need to develop targeted TOC interventions specifically tailored to meet the needs of this population. To address this gap, we propose to adapt, implement, and evaluate the Home Environmental Skill-Building Program (ESP) to address the needs of PLWD and their care partners during hospital-to-home transitions. Developed and tested in prior NIH-funded randomized controlled clinical trials (grant numbers: R01AG10947 and U01AG013265), ESP is a caregiver skills training program delivered by an occupational therapist for at-home management of self-care deficits and behavioral problems due to dementia. A health equity-centered approach will be used to adapt the ESP intervention protocol to address the needs of care partners whose primary language is not English, a group at greater risk of receiving poorer quality transitional care. Strategies will be shared through which intervention adaptations may address language and cultural barriers, as social determinants of health, towards ameliorating health disparities during care transitions experienced by PLWD and care partners for whom English is not their primary language.

